# Assessment of the link between in utero exposure to 2-aminoanthracene (2AA) and type-1 diabetes (T1D)

**DOI:** 10.1186/s40200-017-0286-6

**Published:** 2017-01-28

**Authors:** Christopher A. Mays, Daniel A. Hunter, Wilson Yau, Worlanyo E. Gato

**Affiliations:** 1 Assistant Professor of Biochemistry, Department of Chemistry, Georgia Southern University, Statesboro, GA 30458 USA; 2 Department of Pathology, College of Veterinary Medicine, The University of Georgia, Athens, GA 30602 USA

**Keywords:** Type-1 diabetes (T1D), 2-aminoanthracene (2AA), Cytokines, Pancreas, Insulin levels

## Abstract

**Background:**

A recent diabetes report revealed an increased incidence in diabetes including type 1-diabetes (T1D). The increase in the numbers of T1D incidences are thought to be related to environmental reasons such as the exposure to environmental chemicals including arylamine 2-aminoanthracene (2AA). T1D is an autoimmune disease of the pancreatic islet in which insulin-producing beta cells are destroyed by auto-reactive T-cells and monocytic cells.

**Methods:**

The purpose of this study is to examine the extent to which 2AA exposure contributes to T1D. Three groups of pregnant Sprague Dawley dams ingested various concentrations of dietary 2AA from gestation through the postnatal period. A select number of cytokines and adipokines previously noted to play a significant role in inflammatory response were analyzed in the pancreas of the pups for alteration. The anatomy of the pancreas was also evaluated to determine any histological changes.

**Results:**

Results showed over-expression of pro-inflammatory protein IL-6. Up-regulation of humoral genes IL-7 and IL-21 were also noted. Pathologic characterization showed no significant changes. Moreover, serum total protein was significantly reduced in exposed groups. Elevated serum glucose concentration seems to correspond to slightly lower insulin levels in serum. Cumulative neonatal weight gain analysis showed no major alterations between the control and gestationally-exposed rats.

**Conclusion:**

It appears that systemic effects of 2AA ingestion were mild in the neonates. Further assessments of pups who lived longer than two weeks could be a useful way to measure the progression and possibly further support our hypothesis that 2AA can lead to systemic effects that are indicative of inducing T1D.

## Background

Diabetes remained the 7th leading cause of death in 2010 due to the number of death certificates listing it as the underlying cause of death [1]. The incidence of diabetes in 2012 was 1.7 million and in 2010 it was 1.9 million. In the 2008–2009 report by the Centers for Disease Control and Prevention, an estimated 18,436 people younger than 20 years in the U.S. were newly diagnosed with type-1 diabetes (T1D) annually. This estimation is based on the demographics of the US population taking into account socioeconomic state, urbanization, age, and gender contributions to diabetes prevalence [2].

T1D is an unpreventable, autoimmune disease of the islet of Langerhans by which insulin-producing beta cells are destroyed by auto-reactive T-cells and monocytic cells [3]. The pancreas contains 5 types of cells: α, β, δ, ε, and PP (pancreatic polypeptide) cells. Eighty percent of the pancreatic cells in rodents are β-cells which are the primary sources of insulin production [4]. Type-1 diabetes is a progressive disease whereby the β cells are gradually destroyed after the manifestation of inflamed islets during an autoimmune response. It is unclear if β-cell apoptosis leads to an autoimmune response or if an autoimmune response leads to β-cell apoptosis, but it is known that cytokines play an essential role because they recruit immune cells [5].

This disease is triggered in two different ways: during injury/infection to the pancreas or during natural β-cell death through organ remodeling. In an injury or infection to the pancreas, macrophages enter the area and cause an inflammatory reaction. The pro-inflammatory reaction signals macrophages and T cells. The T cells that are products of the immune response attack the β cells that produce insulin. The dysregulation of the excretion of autolytic cells, for reasons unknown, is the cause of continued destruction of the β-cells. Organ remodeling is performed through natural physiological death, which does not cause an inflammatory response. However, if these cells are not excreted properly they can be released, resembling unwanted materials, and produce a pathogen-induced inflammatory response. The cause behind this autoimmune response has not been understood very well [6]. Nevertheless, T1D is a disease by which the pancreas is not able to produce or secrete enough insulin to metabolize glucose within the body and this makes the organism insulin dependent. Clinical research has shown that instances of β-cell destruction are associated with a decline in insulin secretion [7, 8].

Many factors such as obesity, genetic predisposition, and environmental pollutants [9] can cause a person to become more susceptible to developing T1D. Also tumor necrosis factor-alpha (TNFα) and IL-1β, which are pro-inflammatory cytokines are increased in the pancreas of obese mice [7]. An exogenous factor such as exposure to polycyclic aromatic hydrocarbons (PAHs) can catalyze inflammatory events within an organism. For instance, application of a PAH, 2-aminoanthracene (2AA) to the skin amplified dermal inflammation [8].

In this project, we study the correlation between a PAH named 2AA and T1D. 2AA belongs to a class of PAHs and it is a carcinogen employed in the manufacturing of dyes, drugs, inks, and agricultural chemicals. It can be found in emissions from domestic heating, tobacco smoke, and automobiles. It is more common in colder climates and winter seasons due to increased use of heating and coal. This pollutant can be extracted from the yards of homes that live near industrial plants that process creosote or coal [10–12].

It is prudent that we continue to examine all possible causes and the genetic basis of T1D. The purpose of this study is to examine any link between 2AA ingestion in dams and T1D events in the neonates. *We hypothesize that dams that ingest 2AA during gestation will show systemic effects in the offspring that is pro-inflammatory response dependent.*

## Methods

### Study design

Nine timed pregnant dams (Day 1) were purchased from Taconic Hudson, NY and assigned into dose regiments of 0 mg/kg- (control-C), 50 mg/kg- (low dose-LD) and 100 mg/kg-diet (high dose-HD) 2AA to reflect environmental exposure levels. It was previously estimated that 5 mg/week of 2AA consuming 0.0065% daily dietary dose for one week [13]. Furthermore, chronic ingestion of 2AA diet produced toxicological effects in the pancreas of rats [14]. The dams were fed 2AA contaminated diet during the periods of gestation and postpartum. Rat pups were sacrificed two weeks after postpartum. The animals were housed at the Georgia Southern University Animal Facility (1176A Biological Sciences Fieldhouse). This facility is accredited by Association for Assessment and Accreditation of Laboratory Animal Care (AAALAC). The rats were treated according to the principles outlined in the ILAR’s (Institute or Laboratory Animal Research) Guide for Care and Use of Laboratory Animals. Our protocols were reviewed and approved by Institutional Animal Care and Use Committee (IACUC protocol# I13010). We were careful to minimize the number of animals employed in the research as well as minimizing animal discomfort.

### Preparation of diet containing 2AA

The 2AA (CAS# 613-13-8) with 98% purity was purchased from Sigma Aldrich (St. Louis, MO) and used without further purification. The appropriate amount of 2AA was initially mixed with sucrose and shipped to Harlan Laboratories Inc., Madison WI for incorporation into the Global Rodent Diet 2020. The base diet 2020X diet was designed to support gestation, lactation, and growth of the rats. Full details of the composition of the diet can be found on Envigo’s website [15]. 2AA was incorporated into the 2020 diet to ensure even distribution of the compound. A mixture of sucrose and 2AA was premixed with some of the powdered-diet and then mixed with the rest of the diet for even distribution. Approximately 10% of water was added to the diet and then it was pelleted. Heat was not added during the pelleting process. The diet was finally dried at 50 °C for 8 h in order to reduce moisture and possible mold contamination. The diet was then packaged and shipped. Control diet was pelleted similar to 2AA adulterated diet to ensure uniformity in diet preparation.

### Histopathology

Pancreas of rat pups were fixed in 10% neutral buffered formalin for at least 48 h, trimmed, routinely processed for histology, sectioned at 4-μm thickness, and stained with hematoxylin and eosin.

### Serum analysis of glucose, insulin, and total protein

Serum glucose was measured using a colorimetric method developed for the RX Monza (Randox Laboratories Ltd, Crumlin, UK) [16]. The method employs enzymatic oxidation in the presence of glucose oxidase. The produced hydrogen peroxide in the presence of peroxidase reacts with phenol and 4-aminophenazone to yield a red to violet product that has intensity proportional glucose concentration. Samples as well as standards were prepped according to Randox’s Glucose (Glu-Pup - GL3815) kit instructions and assayed at 505 nm.

Serum total protein (TP) was also determined using Biuret Method modified for Randox Monza clinical analyzer instrument (Randox Laboratories Ltd, Kearneysville, WV) [17]. Similar to the glucose assay, TP (TP245, Randox Laboratories Ltd, Kearneysville, WV) samples and standards were prepared according to the guidelines provided by Randox Laboratories.

The concentration of insulin in the serum was quantified using enzyme-linked immunosorbent assay (ELISA) technique. Details of the experimental approach can be found in the Ultra Sensitive Rat Insulin ELISA kit (Cat# 90060, Crystal Chem Inc, Downers Grove IL) and others [18]. Briefly, insulin samples were bound to guinea pig anti-insulin antibody on microplate and immobilized, unbound materials were removed through washing, and substrate solution was added to generate a colored product and measured on a microplate reader (Spectra Max 190, Molecular Devices Corporation, Sunnyvale, CA, USA) at 450 nm and 630 nm.

### Quantification of selected mRNAs by RT-PCR

Gene transcripts that are important in mediating inflammatory and diabetic processes were quantified via quantitative qRT-PCR. The pro-inflammatory cytokines that were examined were TNF-α, IL-1β, and IL-6. Further, genes such as 1 L-21, IL-7, CD68, and CD14, which are important for adaptive immunity, were also quantified. In addition, two essential hormones (adiponectin and leptin) secreted by adipose tissue were also measured. β-Actin was quantified as a housekeeping gene. FASTA mRNA sequences of these mRNA transcripts were obtained from *Rattus norvegicus* using the National Center for the Biotechnology Information (NCBI) database. Forward and reverse primers for the genes were then generated using NCBI Primer-Blast. Primers were bought from Integrated DNA Technologies Inc (IDT), Coralville IA USA.

An iScript cDNA synthesis kit was employed to synthesize cDNAs from total RNA extracted using Rneasy Plus Universal Mini by Qiagen. The cDNAs were combined with primers and SsoFast EvaGreen supermix for the qPCR reaction using β-actin as a control transcript. The product was quantified via a Bio-Rad CFX96TM instrument (Bio-Rad Laboratories Inc.) using the manufacturer’s guidelines. The normalized relative gene expression values were determined via the delta Ct parameter.

### Data analysis

Statistically significant differences in the aggregate pup weights, total protein levels, serum and IL6 amount, glucose, and insulin concentrations in control groups were compared with 2AA exposed groups using analysis of variance (ANOVA). Data were presented as mean ± SE. Significant differences were indicated as either * *P* < 0.05 or ** *P* < 0.01.

## Results

### Effect of 2AA on neonatal weight

**Fig. 1:**
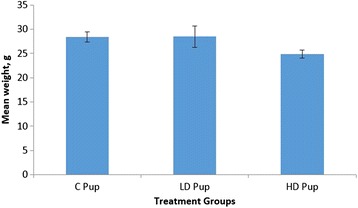
Mean weight of pups (*n* = 12–18) two weeks postpartum. Average weight between control and 50 mg/kg 2AA was similar. However, there was marginal *P* = 0.073671) reduction in the 100 mg/kg group

The body weights of the pups were recorded at the time of birth and up until time of sacrifice (Fig. 1). No significant differences in the weight of pups were observed between the control and low dose animals. However, the high dose group was marginally significantly lighter in weight compared to the control group.

### Analysis of total protein (TP) in the serum

**Fig. 2:**
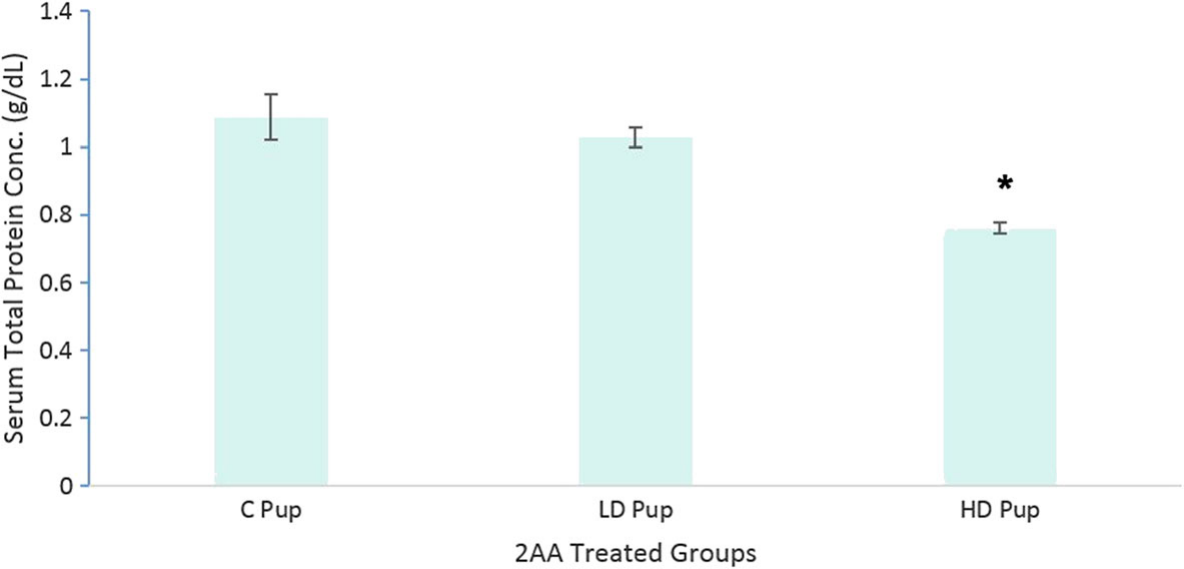
Total protein level (g/dL) ± standard error (SE) assay of the serum of Sprague Dawley rat offspring exposed to 2-AA in utero (n = 4) (*P* < 0.01). Neonates were exposed to control (C – 0 mg/kg); low dose (LD – 50 mg/kg) and high dose (100 mg/kg)-2AA diet

Additional neonatal systemic toxic effects due to *in utero* exposure of 2AA was determined through the serum total protein concentration (Fig. 2). Significant reduction or TP in the high dose group occurred.

### Histological analysis of pancreas

**Fig. 3:**
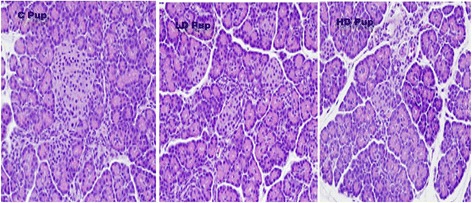
Histopathology pancreatic tissues (n = 7–10). Sprague Dawley dams ingested 0 mg/kg- (C Pup), 50 mg/kg- (LD Pup) and 100 mg/kg-2AA (HD Pup) from gestation through postnatal period. Slides were H&E stained

Histopathologic characterization did not show significant qualitative difference between *in utero* exposed groups and non-treated rats (Fig. 3). This is expected due to the short 2-week interval allowed to provide a physical effect upon the pancreas.

### 2AA effect on select inflammatory genes

**Fig. 4:**
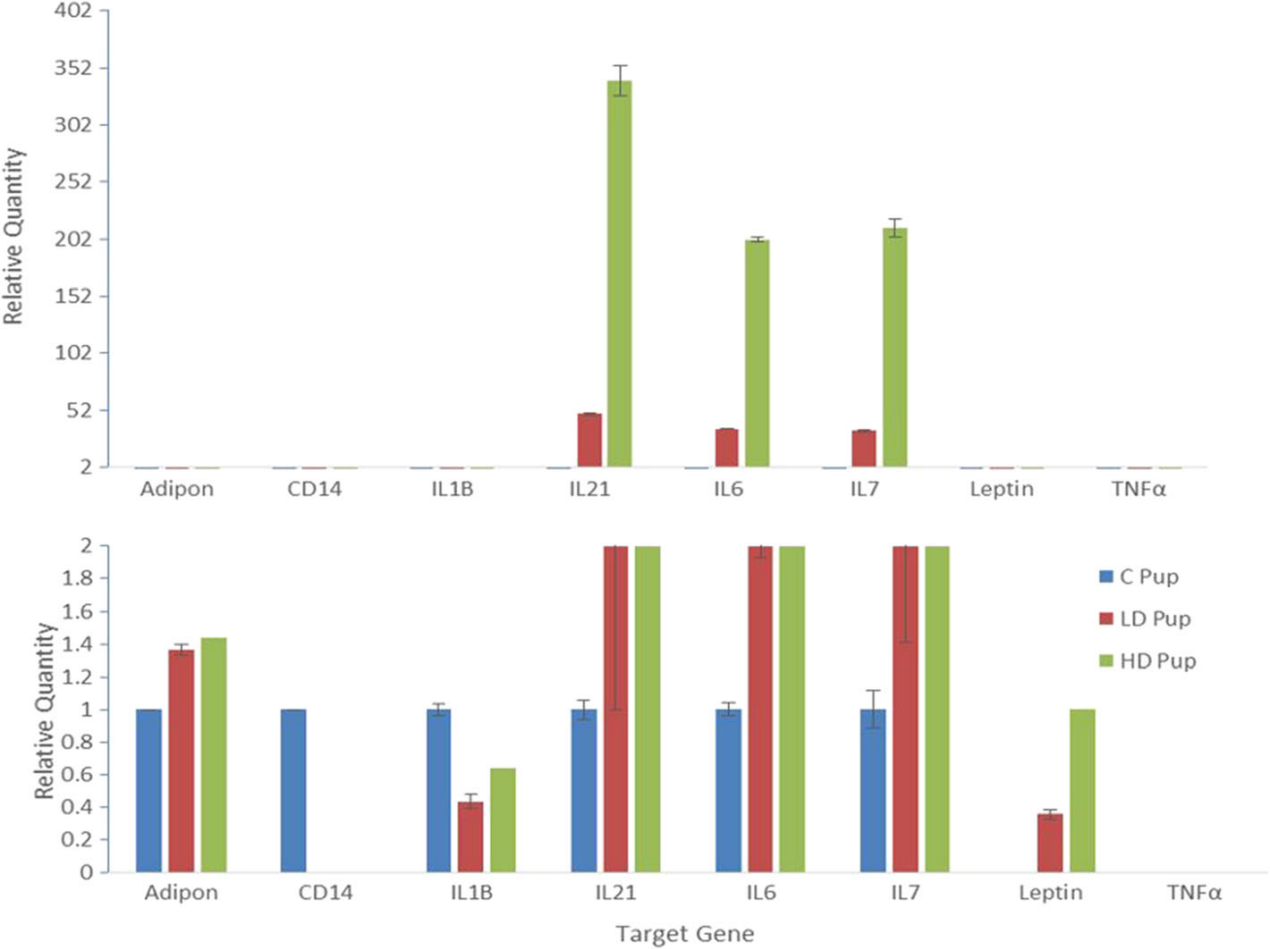
The relative expression (∆Cq) of a select pancreatic mRNAs that play crucial roles in the cellular inflammatory process. Progeny underwent *in utero* exposure to 2AA were 0 mg/kg 2AA diet (C- Control), 50 mg/kg diet (LD- Low Dose) and (100 mg/kg diet (HD- High Dose) from Gestation through 14 days postnatal

The mRNA expression of selected genes (Table 1) were chosen based on their role in the inflammatory process. These were quantified in the pancreas using RT-PCR. The majority of the genes maintained similar expression patterns with the exception of IL-6, an inflammatory cytokine, and IL-21 and IL-7 which are key factors in immune regulation (Fig. 4). These genes are regulated in a dose dependent manner. They are up-regulated in the pups that were exposed to higher concentrations of 2AA *in-utero*.

**Table 1:** Nucleotide sequences designed as forward and reverse primers of each specific gene

Gene Name Primer Sequence		Products size (bp)
Adiponectin	Forward 5’- CCGCTTACATGTATCACTC-3’	248
	Reverse 5’- ATACTGGTCGTAGGTGAAGA - 3’	
Leptin	Forward 5’-CTGTCGTGACTGACTCTATG-3’	588
	Reverse 5’- GCTAAGTGATTTCTCATTCC -3’	
CD68	Forward 5’- AAGTCCTAGTCCAAGCTCTA -3’	311
	Reverse 5’- AGGACACATTGTATTCCACT -3’	
CD14	Forward 5’- CTCAGAATCTACCGACCA -3’	867
	Reverse 5’- ATAGATTGAGCGAGTTTAGC -3’	
IL6	Forward 5’- GGAGTTTGTGAAGAACAACT -3’	77
	Reverse 5’- CTAGGGTTTCAGTATTGCTC -3’	
TNFα	Forward 5’- GAACACCCTGGTACTAACTC -3’	519
	Reverse 5- TAGATAAGGTACAGCCCATC -3’	
IL7	Forward 5’- ATATCAGTGAGGAATTCAATG -3’	134
	Reverse 5’- TAGTCTCTTTAGGAAACATGG -3’	
IL1β	Forward 5’- TGATGTTCCCATTAGACAG -3’	145
	Reverse 5’- TACAAAGCTCATGGAGAATA -3’	
IL21	Forward 5’- GAACAGCTGAAAATCTATGAG -3’	219
	Reverse 5’- CATGTGCCTCTGTTTATTTC -3’	
β-Actin	Forward 5’- CACCCGCGAGTACAAC -3’	720
	Reverse 5’- TACATAGCACAGCTTCTCTTT -3’

### Pup serum analysis

**Fig. 5:**
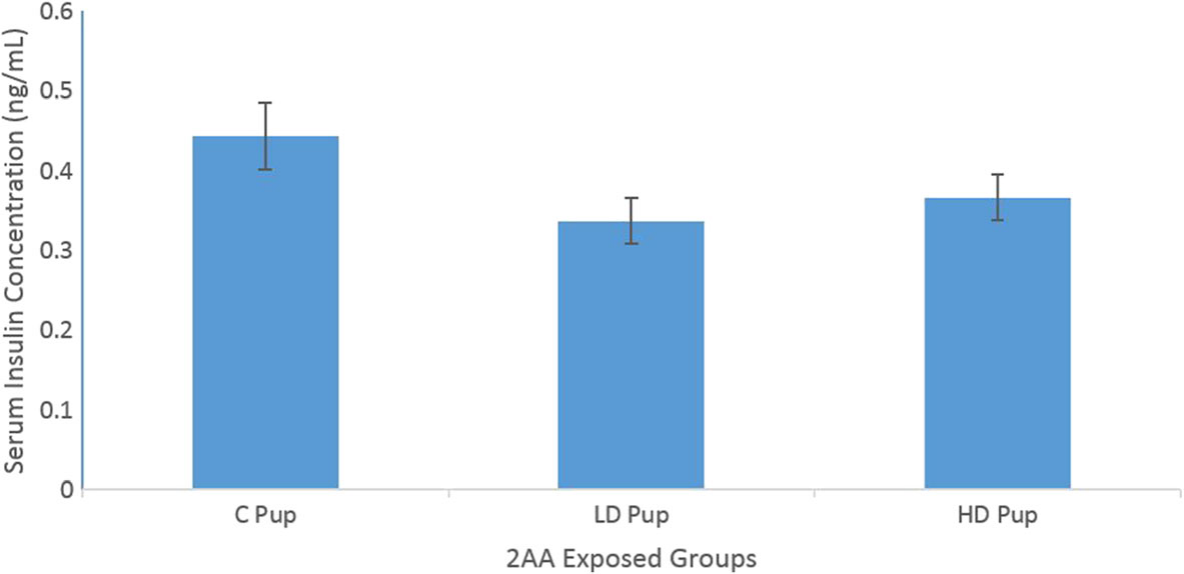
Mean serum insulin concentration of Sprague Dawley rat offspring exposed to 2AA in-utero (*n* = 4). Neonates were systemically exposed to control (C – 0 mg/kg); low dose (LD – 50 mg/kg) and high dose (100 mg/kg)-2AA diet during gestation. Insulin levels were not significantly different though slightly lower in 2AA exposed groups

**Fig. 6:**
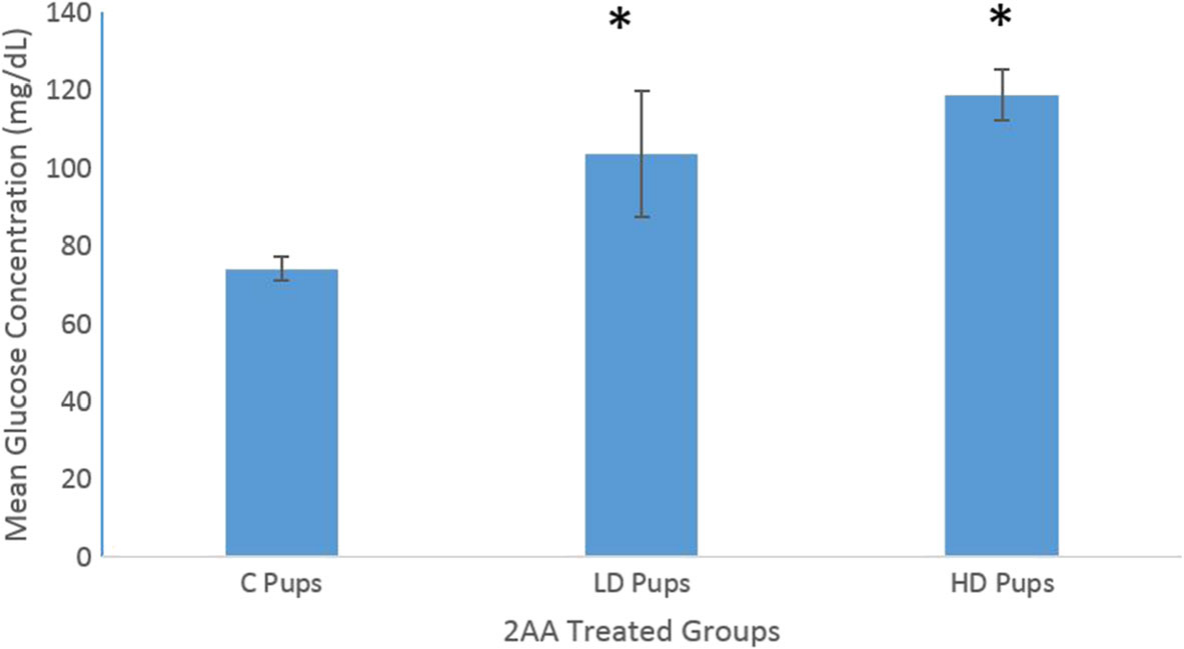
Mean serum glucose concentration of Sprague Dawley rat offspring whose mothers ingested 2AA during gestation through postnatal period. Neonates exposed to 2AA *in utero* had significantly higher (*p* < 0.05) serum glucose levels (*n* = 4). In utero exposure included control (C – 0 mg/kg); low dose (LD – 50 mg/kg) and high dose (100 mg/kg)-2AA diet

**Fig. 7:**
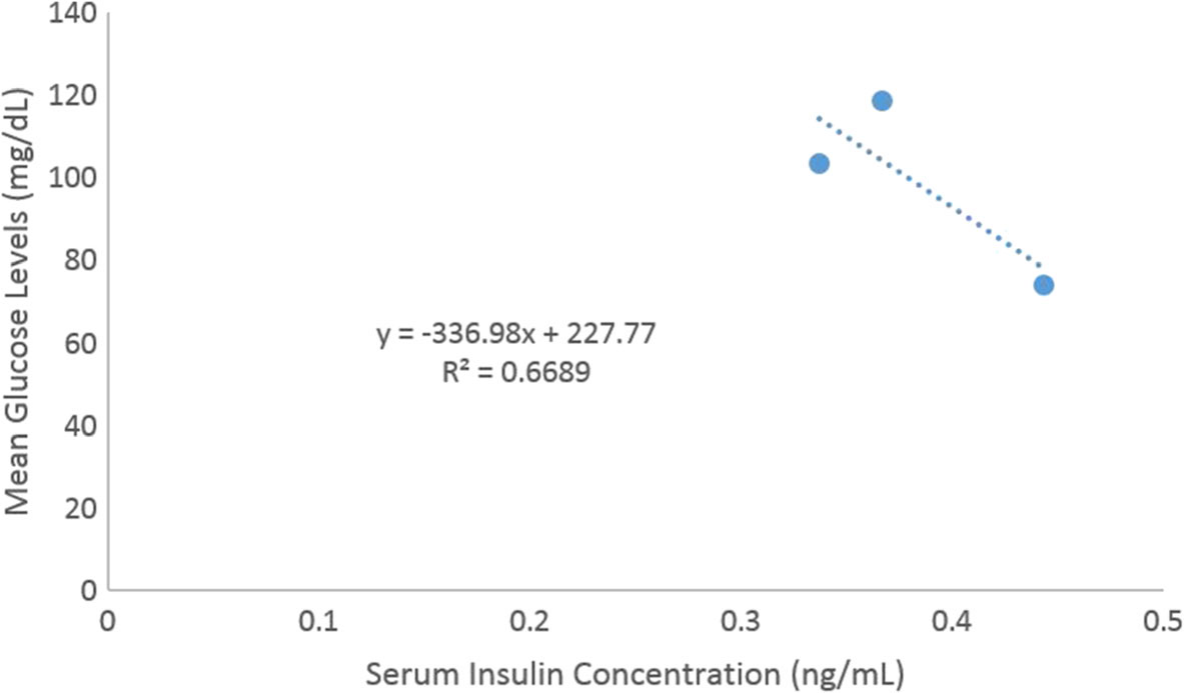
Comparison between mean serum insulin and glucose concentration of Sprague Dawley rat offspring exposed to 2-aminoanthrancene in-utero. Serum samples taken 2 weeks postnatal and postweaning. (*n* = 4)

An evaluation of the pup serum between the *in utero* exposed groups and non-treated rats was performed for glucose and insulin utilizing colorimetric techniques. An assessment of glucose and insulin concentrations enabled the comparison on the systemic effects of exposure to 2AA. The experimental groups do not appear to show any significant difference between their insulin concentrations (Fig. 5). However, there were significant differences between the experimental groups and control groups in assessment of glucose levels (Fig. 6). Insulin concentrations plotted against glucose amounts displayed an inverse relationship (Fig. 7). This might suggest a cellular glucose dependence on insulin levels.

### Validation of gene expression data via protein concentration of IL-6

**Fig. 8:**
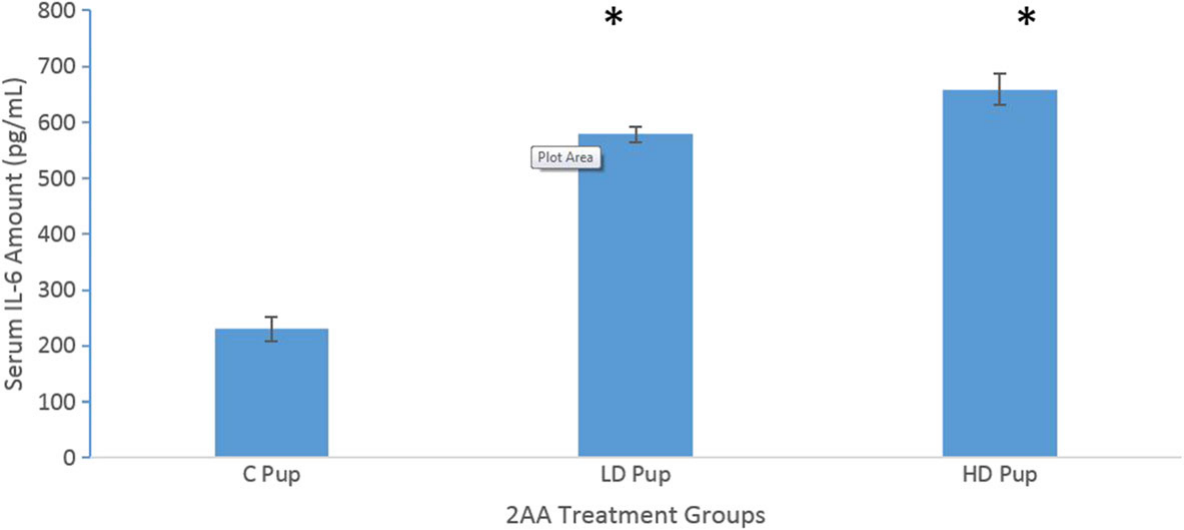
Mean serum IL-6 concentrations of Sprague Dawley rat offspring exposed to 2-AA in-utero. Samples taken 2 weeks postnatal. Experimental groups show a significant increase (*p* < .0.05) in IL-6 concentration

Cytokine IL-6 concentrations were quantified in serum to supplement the gene expression data due to the pleiotropic role that IL-6 plays in signaling pathways of acute phase response genes (Fig. 8). The experimental groups show significantly higher IL-6 concentrations than the control group.

## Discussion

The present study examines whether gestational exposure to 2AA, a PAH, will cause susceptibility in neonates towards the development of T1D. The primary predictor of diabetes within an individual is hyperglycemia [19]. T1D and type-2 diabetes (T2D) can be differentiated by quantifying the presence of ketones or testing the presence of autoantibodies specific to insulin [20]. Insulin is produced in the β cells of the islets of Langerhans. This hormone is known to regulate glucose metabolism [21]. Insulin has three functions as glucose enters the body. It signals the cells of insulin-sensitive peripheral tissues to absorb glucose, it acts on the liver to initiate glycogenesis, and it inhibits the liver from performing gluconeogenesis and glycogenolysis [22].

To examine the effect of 2AA on pups exposed to 2AA *in utero*, TP tests were undertaken. Significant reduction in TP levels within the HD experimental group were noted. Albumins and globulins are the two most abundant proteins present in a mammal and a shift in TP is most likely a shift in the ratio of the two proteins [23]. A high A/G ratio suggest an underproduction of immunoglobulins which is commonly seen in genetic deficiencies [24]. The HD group has a lower concentration of total protein which can be an indication of liver or kidney disorder. Total protein assessments are non-definitive and further tests are needed to aid in determining the toxic effects. However, the assessment is useful in providing preliminary evaluations.

The histological analysis of the pancreas showed a negligible qualitative difference between the control and experimental groups. As previously stated, it takes up 80% of pancreatic beta cells to be destroyed in order for one to be formally diagnosed [7]. The pups in this study were euthanized two weeks post-natal. The progression to type-1 diabetes has yet to be observed, but may it take more than two weeks before structural changes can be detected within the pancreas via histology. On the other hand, functional differences are evident within two weeks, as demonstrated here by the systemic effects in specific gene expressions, glucose levels, and total protein levels.

Results suggest that environmental factors can hinder the action of insulin within the animal. The gene expression data of the rat offspring showed a concentration-dependent mRNA targets due the consumption of 2AA. Cytokines IL-6, IL-21, and IL-7 are up-regulated in rats that consumed the highest concentration of 2AA. There is evidence noting that IL-6 is present in many autoimmune disorders because it can aid in the development of the disease. This development may be induced through IL-6 because it is responsible for initiating acute phase responses, β-cell proliferation, and production of autoantibodies [25]. IL-6 was up-regulated in many diseases such as rheumatoid arthritis, multiple sclerosis, and lupus [26]. However, IL-6 was not proven to be a cause of type-1 diabetes because there were many other proteins that serve as regulatory factors on the pancreatic beta cells. Furthermore, IL-6 was shown to be present in T2D malady, indicating this cytokine’s different regulatory role via divergent mechanisms [26].

IL-21 was also up-regulated. IL-21 is an inflammatory protein that signals via the JAK-STAT signaling pathway utilizing STAT3, similar to IL-6, to drive IG production and proliferation of T and B cells [2]. This finding suggests that there might be a higher immune activity within rodents whose mothers ingested 2AA. Furthermore, there have been studies showing rodents with IL-21 deficiency [2] have fewer and less functional T_H_17 helper cells. These cells are crucial in T1D development. Our results indicate fewer cases of inflamed insulin [3]. Similarly, IL-7 was also up-regulated. IL-7 was shown to be important for lymphoid cell survival and it plays a part in the development of T and B cells. It binds to hepatocyte growth factor to serve as a B cell growth stimulating factor [27]. Previous studies introduced IL-7 antibodies into non-obese diabetic mice and results appeared to reverse the disease [28]. Consequently, IL-7 is also responsible for stimulating expression of T-helper cells as well [28]. Though the expression of key inflammatory genes were observed, it cannot be said at this point that 2AA exposure causes T1D in rodents. This is primarily due to the fact that the mechanism of the effects of 2-aminoanthracene has yet to be elucidated. 2AA is within a class of arylamines and it is known that they are mutagens that induce changes upon DNA. However, a previous study showed the effects upon pancreatic islets due to the prolonged exposure of 2AA, approximately 80 days. The physical effects were the production of intracellular vacuoles, destruction of proximal acinar cells, and a reduced detection of insulin within these cells. Studies have shown that vacuole formation may precede autophagic cell death through the upregulation of Atg5 which acts upon Fas-associated protein with death domain. This is a possible mechanism as to how 2AA can lead to the pancreatic islet destruction [14, 29].

On the contrary, measurement of serum glucose displayed a stronger support for the hypothesis under study. Significant differences appeared between the control and gestational-exposed groups. One can infer that prolonged exposure may lead to hyperglycemic conditions. Clinical research has revealed that IL-6 can induce glucagon secretion via direct stimulation of the pancreas’ α-cells or the brain during stimulation of the sympathetic nervous system [30]. In other words, the induction of IL-6 dependent glucagon dependent is also directly correlated with the epinephrine produced. It is likely the increased concentration of glucose IL-6 expression is due to the two possible pathways for glucagon secretion via IL-6 [30]. In addition, the LD group apparently has a slightly higher concentration of glucose than the control and HD animals.

With regards to the serum insulin levels, the experimental rodents exhibited slightly lower concentrations than the control group, although the reduction in insulin amounts was not significant. Regarding lower insulin concentration, hyperglycemic conditions can be the consequence because higher levels of glucose may be due to low concentrations of insulin in the cell. The findings from the present study are similar to a previous investigation that assessed chronic exposure of 2AA [14]. In that study, no significant change in the architecture of the pancreas or increased glucose levels were observed until day 63 after continuous 2AA dietary ingestion.

### Conclusions

The present study examined whether gestational exposure to a PAH named 2AA could lead to increased susceptibility to T1D. Results indicate an over-expression of pro-inflammatory and adaptive immunity gene transcripts. Moreover, serum total protein was significantly reduced in exposed groups. Elevated serum glucose concentration seems to correspond to slightly lower insulin levels in serum. Cumulative neonatal weight gains and histological analysis showed no major alterations between the control and gestational-exposed rats. It appears that systemic effects of 2AA ingestion were mild in the neonates. Further assessments of pups who lived longer than two weeks could be a useful way to measure the progression and possibly further support our hypothesis that 2AA can lead to systemic effects that are reflective of inducing T1D.
